# *Cinnamomum zeylanicum*,* Origanum vulgare*, and* Curcuma longa* Essential Oils: Chemical Composition, Antimicrobial and Antileishmanial Activity

**DOI:** 10.1155/2019/2421695

**Published:** 2019-01-15

**Authors:** Amanda Mara Teles, Taynan Dulce da Silva Rosa, Adenilde Nascimento Mouchrek, Ana Lucia Abreu-Silva, Kátia da Silva Calabrese, Fernando Almeida-Souza

**Affiliations:** ^1^Laboratório de Controle de Qualidade de Alimentos e Água, Universidade Federal do Maranhão, 65065-545 São Luís, MA, Brazil; ^2^Rede Nordeste de Biotecnologia, Ponto Focal Maranhão, São Luís, Brazil; ^3^Mestrado em Ciência Animal, Universidade Estadual do Maranhão, 65055-310 São Luís, MA, Brazil; ^4^Laboratório de Imunomodulação e Protozoologia, Instituto Oswaldo Cruz, Fiocruz, 21040-900 Rio de Janeiro, Brazil

## Abstract

The resistance mechanisms of bacteria and protozoans have evidenced the need of discover new compounds with potential pharmaceutical activity against pathogenic microorganisms. Medicinal plants have been for centuries a promising alternative as sources of new drugs. The objective of this work was to evaluate the chemical composition, antimicrobial and antileishmanial activities of* Cinnamomum zeylanicum*,* Origanum vulgare*, and* Curcuma longa* essential oils. Chemical analysis was performed by gas chromatography-mass spectrometry. Antimicrobial activity was performed by disk diffusion and minimum inhibitory concentration (MIC) test. Antileishmanial activity was performed against antipromastigote and intracellular amastigote of* Leishmania amazonensis*. Cytotoxic and nitrite production were realized in BALB/c peritoneal macrophages. The major compounds of the essential oils were cinnamic aldehyde (46.30%) in* C. zeylanicum*, cis-p-menth-2-en-1-ol (33.88%) and linalyl acetate (13.90%) in* O. vulgare*, and turmerone (55.43%) in* C. longa*. The MIC showed significant antimicrobial activity of* C. longa* essential oil against* S. aureus* (83.3 ± 14.43 *µ*g/mL). Antipromastigote activity showed IC_50_ values >500 *µ*g/mL to* C. zeylanicum*, 308.4 ± 1.402 *µ*g/mL to* O. vulgare*, and 405.5 ± 1.119 *µ*g/mL to* C. longa* essential oil. Activity against intracellular amastigote of* L. amazonensis *showed IC_50_ of 63.3 ± 1.369 *µ*g/mL and cytotoxic was not observed, resulting in selectivity index higher than 15.79 to parasite.* C. longa* essential oil decreased nitrite production in peritoneal macrophages, but not in* Leishmania-*infected cells. The chemical composition of the three essential oils is directly associated to its potential biological action, as the antimicrobial activity.* C. longa* presented a potent antileishmanial activity against promastigote and intracellular amastigote of* L. amazonensis*, although this activity is not linked to nitric oxide, since* C. longa* essential oil inhibits its production.

## 1. Introduction

Hospital-acquired infections are directly linked to Gram-positive pathogens as* Staphylococcus aureus*, and Gram-negative pathogens as* Escherichia coli* and* Pseudomonas aeruginosa*. It is estimated that in the United States in the 1990s hospital infections cost $4.5 billion and contributed to more than 88,000 deaths [1]. The main treatment adopted to combat hospital infections is the indiscriminate use of antimicrobials that generated methicillin-resistant* S. aureus*. Frequent use of antibiotics is cited as the cause of various bacteria resistance to a range of commonly available antibiotics, especially penicillin [2].

Drugs used during treatment of infections are generally associated with adverse effects on the patient, including hypersensitivity, hepatotoxicity, and nefrotoxicity. The treatment for leishmaniasis, a complex of disease that affects millions of people around the world, is one of the treatments where cases of resistance and toxicity are constantly reported [3–5]. In the face of these side effects and the resistance that pathogenic microorganisms have acquired, research has been focused with attention on essential oils, extracts, and biologically active compounds isolated from plants species used in traditional medicine.

The genus* Cinnamomum* includes approximately 250 species; among these is* Cinnamomum zeylanicum* Blume (Lauraceae) commonly called cinnamon. It is widely used as a seasoning in cooking because of its characteristic flavor. It is cultivated mainly in countries like India, Sri Lanka, and China. Extracts, essential oils, and cinnamon isolates have excellent applications in food, cosmetics, and pesticides, due to antimicrobial, antioxidant, and antifungal properties [6, 7]. In traditional medicine, it has wide use as a remedy for indigestion, diabetes, acne, respiratory, and urinary problems, etc. [8].

Turmeric (*Curcuma longa* L.) is a plant native to Southeast Asia that belongs to the Zingiberaceae family. As a powder called saffron, it has been used continuously as a seasoning in vegetarian and nonvegetarian food preparations and also has digestive properties. Its pyriform rhizomes, often short and branched, are home remedies used in folk medicine. The therapeutic properties of turmeric or its compounds include antimicrobial [9], antifungal, and antioxidant [10] activity. Antileishmanial activity of molecules isolated from* C. longa* has been described in trial assays to promastigote forms, but there is no data about its effectiveness against intracellular amastigote, the form found in mammalian hosts [11].

Oregano or marjoram (*Origanum vulgare* Linn.) is the most variable species of the genus* Origanum*, characterized by morphological and chemical diversity. Used worldwide since ancient times in traditional and popular medicine, oregano is spread all over the world and is particularly abundant in the Mediterranean, Eurasian, and North African area. Several studies have shown that the plant has a wide variety of secondary metabolites, most of them phenolic compounds such as flavonoids, terpenoids, phenolic acids and alkaloids, and fatty acids, among others, which are the main components responsible for its action [12].

Due to the wide use of these plants, associated with the need to discover new compounds with potential pharmaceutical activity against pathogens, this work aimed to evaluate the chemical composition and antimicrobial and antileishmanial activities of* Cinnamomum zeylanicum*,* Origanum vulgare*, and* Curcuma longa* essential oils.

## 2. Material and Methods

### 2.1. Plant Material

The leaves of* Cinnamomum zeylanicum* were collected in the city of São Luis, Maranhão, Brazil. The taxonomic identification was made by Ana Zelia Silva in the Seabra Ático Herbarium of the Department of Botany of the Universidade Federal do Maranhão, registry number 1153. Aerial parts of* Origanum vulgare* and* Curcuma longa* rhizome were purchased in the Central Market of São Luís. All three plants were dried for 48 hours and sprayed in an electric knife mill at the Laboratório de Controle de Qualidade de Alimentos e Água of the Universidade Federal do Maranhão.

### 2.2. Essential Oils Extraction

The essential oils were extracted by hydrodistillation using Clevenger system. A quantity of 100g of dry leaves diluted in water at a ratio of 1:10 was boiled at 100°C for 3 hours. The oil was dried over anhydrous sodium sulfate and kept in an amber bottle under refrigeration [13]. For* in vitro* biological assay, the essential oils were dissolved in dimethylsulfoxide (DMSO) at 100 times the highest concentration of use and subsequently diluted in an appropriate medium to a final concentration of less than 1% of DMSO.

### 2.3. Gas Chromatography-Mass Spectrometry Analyses (GC-MS)

The essential oils under study were dissolved in ethyl acetate 1 mg/mL and analyzed on Shimadzu QP 5000 gas chromatograph with ZB-5 ms capillary column (5% phenyl arylene 95% dimethylpolysiloxane) coupled at 70 eV (40-500 Da) HP 5MS mass selective detector of electronic impact with a transference temperature of 280°C. Chromatographic conditions were volume injection of 0.3 *μ*L of ethyl acetate; helium carrier gas (99.99%); injector temperature: 280°C, split mode (1:10); initial temperature of 40°C and a final temperature of 300°C; initial time 5 min and final time 7.5 min at 8°C/min [14].

### 2.4. Bacterial Culture

*Escherichia coli* (ATCC 25922),* Staphylococcus aureus* (ATCC 12600), and* Pseudomonas aeruginosa* (ATCC 27853) strains were cultured in Brain Heart Infusion (BHI) broth for 24 h at 37°C and diluted to 10^8^ CFU/mL following the MacFarland scale [15].

### 2.5. Antimicrobial Assays

Disk diffusion tests were performed with filter paper impregnated with 75 *μ*L of essential oil placed on the surface of Mueller-Hinton agar seeded with 100 *μ*L of each bacterium. Gentamycin, 10*μ*g per disk (Laborclin, Brazil), was used as reference. The plates were incubated at 35°C and after 24 hours the inhibition halo was measured with a millimeter ruler [16]. The minimum inhibitory concentration (MIC) was determined using the broth dilution methodology and performed in triplicate with the same bacterium used in disk diffusion test. An aliquot of the essential oil prepared in DMSO was transferred to a test tube containing BHI broth. Serial dilutions of essential oils were then performed resulting in concentrations of 5–2000 *μ*g/mL. Amoxicillin, gentamycin, and polymyxin B (0.015 a 128 *μ*g/mL) were used as reference. Microbial suspensions containing 1.5x10^8^ CFU/mL of the bacteria were added at each concentration of essential oil or antibiotic drug and incubated at 35°C for 24 h. Tubes without bacteria were used as control of broth sterility and bacterial growth. The MIC was defined as the lowest concentration which visibly inhibited bacterial growth observed by the absence of visible turbidity. BHI broth was subjected to the inoculum microbial seeding test on the surface of plate-count agar to confirm growth inhibition [17].

### 2.6. Parasites

*Leishmania amazonensis* (MHOM/BR/76/MA-76) promastigote forms were cultured at 26°C in Schneider's Insect medium (Sigma, USA) supplemented with 10% fetal bovine serum (Gibco-USA), 100U/mL of penicillin (Gibco, USA), and 100 *μ*g/mL of streptomycin (Sigma, USA) [18]. Cultures with a maximum of seven* in vitro* passages were used.

### 2.7. Animals

Female BALB/c mice, 4–6 weeks old, were purchased from Instituto de Ciência e Tecnologia em Biomodelos of Fundação Oswaldo Cruz, Rio de Janeiro, and maintained in accordance with National Council for Control of Animal Experimentation (Conselho Nacional de Controle de Experimentação Animal–CONCEA). The local Ethics Committee on Animal Care and Utilization approved all procedures involving the animals (CEUA/IOC – L053/2016).

### 2.8. Cell Culture

Peritoneal macrophages were obtained from BALB/c mice elicited with 3 mL 3% thioglycolate for 72 h, and maintained in RPMI 1640 (Sigma, USA) supplemented with 10% fetal bovine serum, penicillin (100 U/mL), and streptomycin (100 *μ*g/mL), at 37°C and 5% CO_2_ [18].

### 2.9. Antipromastigote Assay

*L. amazonensis* promastigote forms from a 2-4-day-old culture were placed in 96-well plates with different concentrations of essential oils (3.9–500 *μ*g/mL) or amphotericin B (0.01–2.5 *μ*g/mL), at a final volume of 100 *μ*L per well for 72 h. Wells without parasites were used as blanks and wells with parasites and DMSO 1% were used as controls. Parasite viability was evaluated by the modified colorimetric method with tetrazolium-dye 3-(4,5-dimethylthiazol-2-yl)-2,5-diphenyltetrazolium bromide (MTT) [19]. 10 *μ*L of MTT (5 mg/mL) was added to each well and after five hours 150 *μ*L of DMSO was added to dissolve the formazan crystals. Absorbance was read on a spectrophotometer at a wavelength of 570 nm. The data was normalized, with absorbance value of control used as 100%, and the results were used to calculate the 50% inhibition of parasite growth (IC_50_).

### 2.10. Cytotoxicity Assay

Peritoneal macrophages were cultured in 96-well plates (5×10^5^ cells/mL) with different concentrations of essential oils (7.8–1000 *μ*g/mL) or amphotericin B (0.07–10 *μ*g/mL) up to a final volume of 100 *μ*L per well, at 37°C and 5% CO_2_. Wells without cells were used as blanks and wells with cells and DMSO 1% only were used as controls. After 24 h, the cells were analyzed by MTT colorimetric method [19]. Briefly, 10 *μ*L of MTT (5 mg/mL) was added to each well and after two hours then the medium was discarded, and 100 *μ*L of DMSO was added to dissolve the formazan crystals. Absorbance values were normalized, with absorbance value of control used as 100%, and the results were used to calculate the 50% cell cytotoxicity (CC_50_).

### 2.11. Activity against Intracellular Amastigote of* L. amazonensis* and Selectivity Index (SI)

Peritoneal macrophages from BALB/c were cultured in 24-well plates (5x10^5^ cells/well), with coverslips, at 37°C and 5% CO_2_. The cells were infected with* L. amazonensis* promastigote forms, 10:1 parasite/cell, for 6 h and afterwords washed to remove noninternalized parasites. The infected cells were treated with different essential oil concentrations (62.5–500 *µ*g/mL) for 24 h. The coverslips with the infected and treated cells were fixed with Bouin, stained with Giemsa, and examined by light microscopy. The IC_50_ was calculated from the total of intracellular amastigote from 200 cells. The percentage of infected cells was obtained from the number of infected cells divided by two. The mean number of amastigotes per cell was obtained from the number of intracellular amastigotes in 200 cells divided by the number of infected cells [20]. Amphotericin B was used as the reference drug. The selectivity index (SI) was obtained from the ratio of BALB/c peritoneal macrophages CC_50_ and intracellular amastigote IC_50_.

### 2.12. Nitrite Quantification of Peritoneal Macrophages Treated with* C. longa* Essential Oil

BALB/c peritoneal macrophages (5x10^6^ cells/mL) were treated with* C. longa* essential oil (400 *µ*g/mL) and/or stimulated with* L. amazonensis* (5x10^7^ parasites/mL). After 48 hours, the supernatant was collected and nitrite quantification performed with Griess reagent. 100*µ*L of culture supernatant was added to 100 *µ*L of Griess reagent (50 *µ*L of sulfanilamide 1% in 2.5% H_3_PO_4_ solution and 50 *µ*L of N-(1-naphthyl)ethylenediamine 0.1% solution) in 96-well plates and after 10 min read at 570nm on the spectrophotometer. The nitrite values were obtained from the standard curve of sodium nitrite (1.5–100 *µ*M) [21].

### 2.13. Statistical Analysis

The numerical results were expressed as mean ± standard deviation and were organized into tables or plotted in graphs. The IC_50_ and CC_50_ were obtained from a nonlinear regression curve of concentration log versus normalized response. Comparison between IC_50_ values was performed by one-way ANOVA and Tukey's multiple comparisons test. Analyses were performed with the software GraphPad Prism 7.00 and differences were considered significant when p<0.05.

## 3. Results

### 3.1. Chemical Composition of* C. zeylanicum*,* C. longa*, and* O. vulgare* Essential Oils

**Figure 1:**
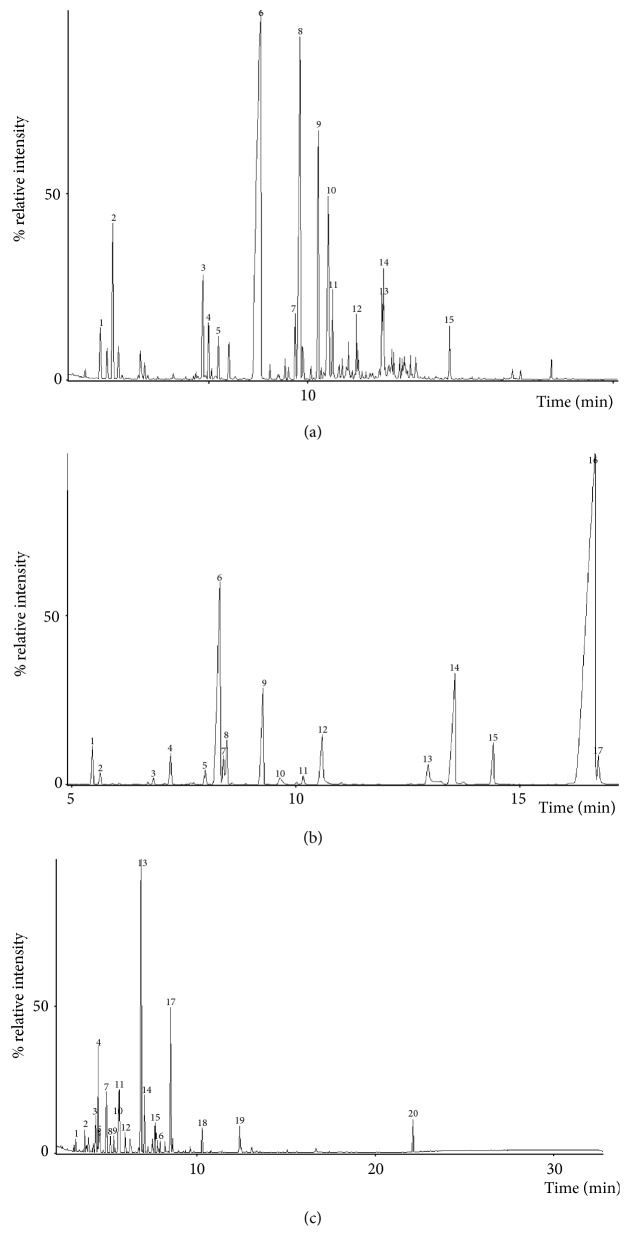
Chromatograms of* Cinnamomum zeylanicum* (a),* Curcuma longa* (b), and* Origanum vulgare* (c) essential oils.

The essential oil yield of* O. vulgare*,* C. zeylanicum*, and* C. longa* was 0.73%, 0.9%, and 3.4%, respectively. GC-MS was used to identify, quantify, and evaluate the chemical compounds present in leaves* C. zeylanicum*, in the aerial parts of* O. vulgare*, and in the rhizome of* C. longa*, as shown in Table 1 and Figure 1. Compounds present in essential oils were enumerated according to elution order and retention time. Fifteen compounds were identified in* C. zeylanicum*, with peaks 6, 8, and 9 being identified as cinnamic aldehyde (46.30%), *α*-copaene (16.35%), and trans-*β*-Caryophyllene (8.26%), respectively, representing its main constituents.* C. longa* essential oil showed 17 compounds with turmerone (55.43%), *β*-turmerone (12.02%), and *γ*-curcumene (6.96) as major compounds.* O. vulgare* essential oil presented 20 compounds, with cis-p-Menth-2-en-1-ol (33.88%) and linalyl acetate (13.90%) identified as the main constituents.

### 3.2. Antimicrobial Activity of* C. zeylanicum*,* C. longa*, and* O. vulgare* Essential Oils

The disc diffusion method showed that the largest halo was observed for* C. zeylanicum* essential oil against* E. coli*, whereas* O. vulgare* and* C. longa* essential oil presented better inhibition against* S. aureus*. The MIC trial showed significant antimicrobial activity of the essential oils studied, with* C. longa* essential showing the better activity against* S. aureus* (Table 2).

### 3.3. Antileishmanial Activity, Cytotoxicity, and SI of* C. zeylanicum*,* O. vulgare*, and* C. longa* Essential Oils

**Figure 2:**
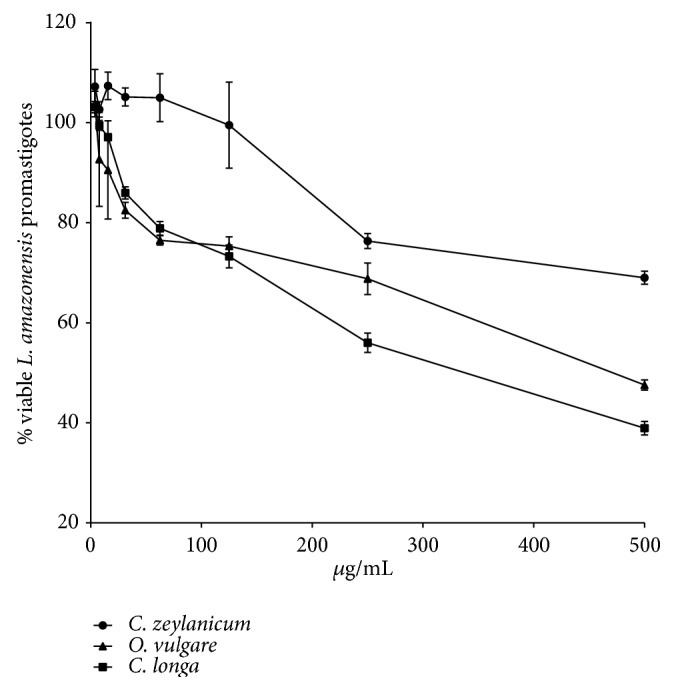
Viability of* Leishmania amazonensis* promastigotes treated for 72 hours with* Cinnamomum zeylanicum*,* Curcuma longa*, and* Origanum vulgare* essential oils.

**Figure 3:**
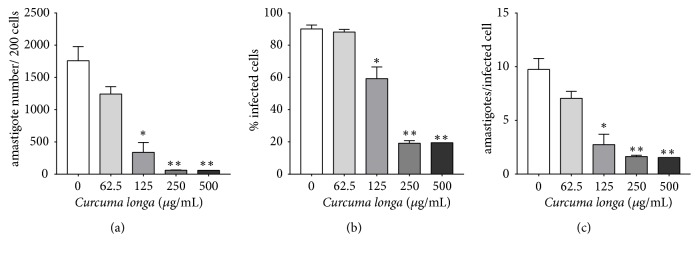
Infection parameters of BALB/c peritoneal macrophages infected with* Leishmania amazonensis* and treated with* Curcuma longa* essential oil. Data represent mean ± standard deviation of two independent experiments realized in triplicate. ^∗^p<0.05 and ^∗∗^p<0.01 when compared with the untreated group by Kruskal-Wallis followed by Dunn's multiple comparisons test.

**Figure 4:**
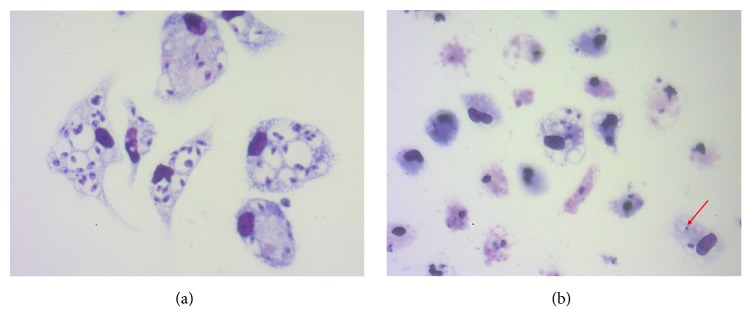
BALB/c peritoneal macrophages infected with* Leishmania amazonensis* and treated with* Curcuma longa* essential oil at 125 *µ*g/mL for 24 hours. (a) Untreated infected macrophages. (b) Treatment decreased intracellular amastigote number and induced loss of intracellular amastigote integrity (red arrow). Images are representative of two experiments realized in triplicate. Giemsa, 40x objective.

Data of the antileishmanial activity, cytotoxicity, and SI are described in Table 3.* C. longa* presented the better antipromastigote activity between the three essential oils, while* C. zeylanicum* showed the worst activity (Figure 2). As the* C. longa* showed the most potent antipromastigote activity between the three essential oils (p<0.0001), the intracellular amastigote activity was performed only with their essential oil, which showed a 4.87-fold decrease in the intracellular amastigote IC_50_ value when compared to the promastigote IC_50_. The analysis of infection parameters showed that at 125 *µ*g/mL there was a statistically significant reduction of all parameters of infection, with decrease of amastigote number in 200 cells and percentage of infected cells of 80.73% and 40.75%, respectively, and mean of amastigotes per cell reduction from 9.74 to 2.74. The decrease in infection parameters was even further reduced at 250 and 500 *µ*g/mL (Figure 3). The decrease in intracellular amastigote number, percentage of infected cells, and mean of amastigotes per infected cell induced by* C. longa* essential oil treatment are observed in Figure 4. None of the three essential oils presented cytotoxicity at the analyzed concentrations. The SI showed that* C. longa* essential oil has activity 15.79-fold more selective to intracellular amastigote of* L. amazonensis* than to BALB/c peritoneal macrophage. The reference drug amphotericin B showed antileishmanial activity and cytotoxicity as expected.

### 3.4. *C. longa* Essential Oil Decreased Nitrite Production in Peritoneal Macrophages

**Figure 5:**
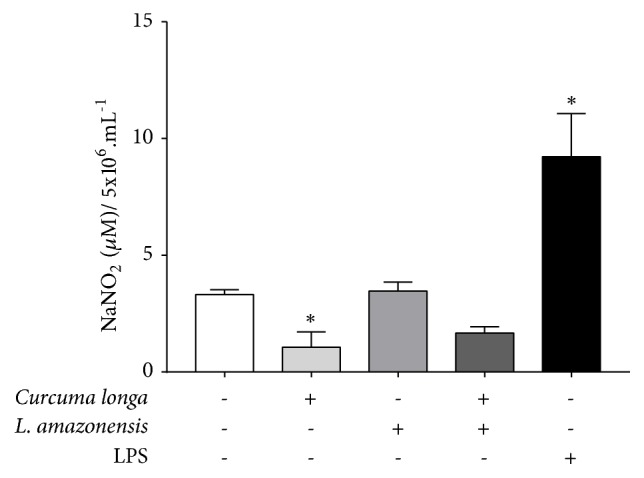
Nitrite quantification in supernatant of BALB/c peritoneal macrophage treated with* Curcuma longa* (400 *µ*g/mL) and stimulated or not with* Leishmania amazonensis*. Data represents media ± standard deviation of experiment realized in sextuplicate; ^∗^p<0.05 when compared with untreated and unstimulated macrophage by Kruskal–Wallis and Dunn's multiple comparison test.

Nitrite quantification in supernatant of BALB/c peritoneal macrophage treated with* C. longa* essential oil (400 *µ*g/mL) showed a significant decrease in nitrite amount from 3.31 *µ*M to 1.06 *µ*M. The same decrease pattern was observed slightly in* L. amazonensis* infected macrophages treated with* C. longa* essential oil when compared with untreated and infected macrophages, from 3.46 *µ*M to 1.66 *µ*M (Figure 5).

## 4. Discussion

In order to analyze the chemical composition and antimicrobial and antileishmanial activity of* C. zeylanicum*,* O. vulgare*, and* C. longa* essential oils, we evaluated the chemical constituents, activity against* E. coli*,* S. aureus*, and* P. aeruginosa*, activity against promastigote and intracellular amastigote of* L. amazonensis*, and cytotoxicity in BALB/c peritoneal macrophage.

Different investigations have identified and quantified several chemical compounds of the essential oils of* C. zeylanicum*,* C. longa*, and* O. vulgare*. The essential oils yield obtained in our extraction was similar to that described in literature [22–24], as well as chemical composition, where cinnamaldehyde is generally the main component of* C. zeylanicum* essential oil [6, 8, 25, 26], and turmerone the major compound of* C. longa* essential oil [27, 28]. On the other hand, there are different chemotypes described to* O. vulgare* revealing different chemical profile, as the chemotype rich in linalool/linalyl acetate with predominant presence of linalyl acetate, the chemotype rich in carvacrol and c-terpinene, and chemotype rich in thymol [29, 30].

The disc diffusion method was used to evaluate the ability of the antibacterial activity of the essential oils of* C. zeylanicum*,* O. vulgare*, and* C. longa* to form inhibition halos against the growth of Gram-positive bacteria (*S. aureus*) and Gram-negative strains (*E. coli* and* P. aeruginosa*). Antimicrobial susceptibility tests of the three essential oils demonstrated active against the standard strains.* C. zeylanicum* essential oil presented larger halos against Gram-positive, and cinnamaldehyde was reported to be responsible for their antimicrobial action [6, 31]. Antimicrobial activity against Gram-positive bacteria has also been reported for* C. longa* [1], and* O. vulgare* has demonstrated efficacy for both Gram-positive and Gram-negative strains [32].

The yield and quality of essential oils vary with genetics, agroclimatic conditions, cultivation techniques, soil conditions, harvest time, etc. Therefore, chemical compositions and major compounds of essential oils vary in different habitats, and their bioactivity is closely associated with changes in their composition. Cinnamic aldehyde, for example, showed MIC ranging from 0.5 to 1000 *μ*g/mL against 20 strains of* P. aeruginosa* [33] and therapeutic potential by inhibiting infections related to biofilm production by* S. aureus* [34]. The turmerone compound found in* C. longa* demonstrated activity against* E. coli* [35] while showed no activity against Gram-positive bacterial strains [36].

Antileishmanial assays showed that* C. zeylanicum* essential oil did not inhibit growth of* L. amazonensis* promastigotes at analyzed concentrations while* O. vulgare* essential oil showed high IC_50_ value. There are few data about antileishmanial activity of these essential oils. An evaluation of* C. zeylanicum* essential oil from Iran against* L. major* promastigotes displayed a significant reduction in the number of parasites [37]. Colombian* O. vulgare* essential oil exhibited activity against promastigotes of* L. panamensis *(IC_50_: 42.23 ± 2.04),* L. braziliensis* (IC_50_:204.36 ± 21.56),* L. major* (IC_50_: >640 ± 0.0), and* L. guyanensis *(IC_50_: 171.8 ± 20.64) [38]. While there are no data about the chemical composition of Colombian* O. vulgare* essential oil that presented antileishmanial activity, its origin indicates a possible difference in chemical composition compared to* O. vulgare* essential oil that we evaluated. In Iranian* C. zeylanicum* essential oil, the presence of 83.47% of (E)-cinnamaldehydein, compared with 46.30% of cinnamic aldehyde observed in our* C. zeylanicum* essential oil, can be a variable responsible for the difference in activity observed between both oils.

*C. longa* essential oil presented the better activity among the three oils, and in literature there are several constituents isolated and related to its antileishmanial activity, with curcumin being the most described. Curcumin has shown an average IC_50_ of 5.3 *µ*M against promastigotes of various leishmanial strains [39]. Curcumin 1, demethoxycurcumin 2, and bis-demethoxycurcumin 3, isolated from the rhizomes of* C. longa*, showed moderate activity against* L. major* promastigote, with IC_50_ of 7.8, 14.1, and 21.5 *µ*g/mL, respectively [40]. Synthetic derivatives of curcumin were ten times more efficient than the original curcumin against* L. amazonensis* promastigotes [41]. Four curcuminoid analogs showed IC_50_ values less than 5 mM to promastigotes of* L. major* and axenic culture of* L. mexicana* amastigotes, with the majority presenting values higher to amastigote axenic than promastigote [42].

As most of the published studies with curcuminoids isolated from* C. longa* against* Leishmania* have been performed against the promastigote form, it is not clear if the activity described in the literature reflects the real leishmanicidal properties of these molecules [11]. Thus, we proceeded to the activity against infected macrophages, a more appropriate model to demonstrate the antileishmanial potential* in vitro*.

First, we performed the cytotoxic analysis against BALB/c peritoneal macrophages, where cytotoxicity was not observed at tested concentrations for any of three essential oils. Then, intracellular amastigote activity was performed and showed that* C. longa* essential oil not only maintained the antileishmanial activity observed against promastigote form but also presented higher efficacy against intracellular amastigote. Trans-dibenzalacetone, a synthetic monoketone analog of curcumin, demonstrated antiproliferative effect on the intracellular amastigotes of* L. donovani* (IC_50_ 7.43 ± 1.88 *μ*g/mL) that also was significantly lower than the IC_50_ value determined for promastigotes (17.80 ± 1.42 *μ*g/mL) [43]. Selectivity index demonstrated the selective antileishmanial activity of* C. longa* essential oil for intracellular amastigote with similar value of trans-dibenzalacetone, SI value of 15.34 for intracellular amastigotes against the BALB/c mouse cell line J774A.1 [43].

The enhancement of activity observed by* C. longa* essential oil against intracellular amastigote suggests that antileishmanial activity may be related to not only direct action against the parasite, but also indirect mechanism. Mechanisms of direct action on* Leishmania* have already been described for the analog of curcumin trans-dibenzalacetone. It alters the general ultrastructure and the mitochondrial physiology of the parasite and triggers apoptotic cell death in* L. donovani* [43]. Macrophage activation is one of the more important mechanisms of indirect action, as induction of reactive oxygen species and reactive oxygen nitrogen species. Several plant materials have antileishmanial activity associated with induction of macrophage activation [18, 44], especially inducing nitric oxide (NO) [21]. Therefore, we evaluated the nitrite production, an indirect measurement to quantify NO, in supernatant of BALB/c peritoneal macrophages infected or not with* L. amazonensis* and treated with* C. longa* essential oil.

Nitrite quantification showed that* C. longa* essential oil inhibited NO production in peritoneal macrophages. The anti-inflammatory activity of* C. longa* (turmeric) has been described for a long time [45], and inhibition of NO production has already been described in a macrophage cell RAW 264.7 stimulated by LPS [46, 47]. Altough we observed an increase of nitrite in peritoneal macrophages stimulated with* L. amazonensis* and treated with* C. longa* essential oil when compared with unstimulated and treated cells, there was no significant enhancement in its NO production due to treatment. Indeed, there is a description of curcumin that overcomes the inhibitory effect of nitric oxide on* L. major* and* L. donovani*. As an antioxidant, curcumin is capable of blocking the action of both NO and NO congeners on the* Leishmania* parasite by acting on S-nitroso-N-acetyl-D,L-penicillamine (SNAP) and DETANONOate, which release NO, 3-morpholino-sydnonimine hydrochloride (SIN-1), which releases NO and superoxide, and peroxynitrite, which is formed from the reaction of NO with superoxide [48]. The inability of inducing NO production in BALB/c peritoneal macrophages reveals that probably there are other possible mechanisms involved in intracellular amastigote activity of* C. longa* essential oil which should be elucidated in further studies.

## 5. Conclusions

The major compounds in the essential oils were cinnamic aldehyde (*C. zeylanicum*), turmerone (*C. longa*), and cis-p-Menth-2-en-1-ol (*O. vulgare*). All three essential oils exhibited antimicrobial action, highlighting* C. longa* essential oil against* S. aureus*. Antileishmanial activity against promastigote forms of* L. amazonensis* was observed to* C. longa* essential oil, but not to* C. zeylanicum* and* O. vulgare* essential oil.* C. longa* essential oil activity against intracellular amastigote is the first description of activity against the parasite form found in mammal host, ensuring their potential antileishmanial activity.* C. longa* essential oil did not induce NO production in BALB/c peritoneal macrophages, suggesting that intracellular amastigote activity may be related to other mechanisms.

## Figures and Tables

**Table 1:** Compounds identified in essential oils from leafs of* Cinnamomum zeylanicum*,* Curcuma longa*, and *Origanum vulgare* by CG-MS.

**Peak** ^**a**^	*Cinnamomum zeylanicum*			*Curcuma longa*			*Origanum vulgare*		
	**t**_**RET**_ ^**b**^	**Compounds**	%**A** ^**c**^	**t**_**RET**_	**Compounds**	%**A**	**t**_**RET**_	**Compounds**	%**A**

1	3.207	*α*-pinene	1.47	5.464	*α*-pinene	1.15	3.221	*α*-pinene	0.80
2	3.618	Benzaldehyde	4.16	5.638	Myrcene	0.37	3.733	Bicyclo[3.1.0]hexane	1.73
3	6.568	3-phenylpropionaldehyde	2.95	6.823	Vinyl propionate	0.20	4.330	(+)-4-Carene	3.08
4	6.748	Borneol	1.06	7.206	p-Cymene	1.01	4.469	p-Cymene	8.29
5	7.077	*α*-terpineol	0.87	7.206	Bisabolone	0.55	4.533	Cyclohexene	1.23
6	8.459	Cinnamic Aldehyde	46.30	8.304	*β*-Turmerone	12.02	4.561	*β*-Phellandrene	2.73
7	9.593	3-Phenyl-1-propanol	1.46	8.385	1,8-cineole	1.01	4.954	p-Menth-2-en-1-ol	4.62
8	9.744	*α*-Copaene	16.35	8.461	Camphor	1.24	5.171	1,4-cyclohexadiene	1.21
9	10.346	trans-*β*-Caryophyllene	8.26	9.267	*α*-Terpineol	4.13	5.361	cis-Sabinene hydrate	1.29
10	10.669	(e)-cinnamyl acetate	7.54	9.645	Terpinolene	0.43	5.598	Terpinolene	3.11
11	10.809	*α*-Humulene	2.16	10.167	*α*-Zingiberene	0.29	5.651	1,6-octadien-3-ol	5.69
12	11.593	delta-cadienene	1.42	10.587	*β*-Sesquiphellandrene	2.67	5.996	trans-Sabinene hydrate	1.59
13	12.422	(-)-Spathulenol	2.09	12.945	*β*-Caryophyllene	1.00	6.877	cis-p-Menth-2-en-1-ol	33.88
14	12.481	Caryophyllene oxide	2.80	13.544	*γ*-Curcumene	6.96	7.079	3-Cyclohexen-1-ol	5.26
15	14.645	benzyl benzoate	1.12	14.397	ar-Curcumene	1.58	7.669	(+)-*α*-Terpineol	2.61
16	–	–	–	16.664	Turmerone	55.43	7.817	Carvacrol methyl ether	0.94
17	–	–	–	16.739	*β*-Sesquiphellandrene	1.10	8.543	Linalyl acetate	13.90
18	–	–	–	–	–	–	10.307	Thymol	2.41
19	–	–	–	–	–	–	12.400	trans-*β*-Caryophyllene	2.46
20	–	–	–	–	–	–	22.115	1H-Cycloprop(E)azulen-7-ol	3.16

**a:** peak number according to the order of column elution. **b: **retention time (minutes) of the compounds in column. **c: **percentage of normalized area which indicates the relative distribution of the compounds in the sample.

**Table 2:** Diameters of inhibition zones and minimum inhibitory concentration of different bacteria culture after 24 hours of treatment with *Cinnamomum zeylanicum*,* Curcuma longa*, or* Origanum vulgare* essential oil.

	Compounds	Bacteria strain		
		*Escherichia coli*	*Staphylococcus aureus*	*Pseudomonas aeruginosa*

Inhibition zones (mm)	*Cinnamomum zeylanicum*	15.00 ± 1.000	14.67 ± 0.577	10.33 ± 0.577
	*Curcuma longa*	12.67 ± 0.577	15.33 ± 0.577	9.66 ± 0.577
	*Origanum vulgare*	14.67 ± 0.577	15.67 ± 0.577	12.00 ± 1.000
	gentamycin	14.33 ± 0.577	20.67 ± 0.577	16.67 ± 0.577

MIC (*μ*g/mL)	*Cinnamomum zeylanicum*	133.3 ± 14.43	216.7 ± 28.87	550.0 ± 0.00
	*Curcuma longa*	216.7 ± 28.87	83.3 ± 14.43	383.3 ± 57.74
	*Origanum vulgare*	266.7 ± 28.87	166.7 ± 28.87	483.3 ± 28.87
	amoxicillin	16.0 ± 0.00	8.0 ± 0.00	n.d.
	gentamycin	n.d.	2.0 ± 0.00	n.d.
	polymyxin B	n.d.	n.d.	16.0 ± 0.00

To determine the inhibition zones, 75*μ*L of each essential oil was used in the disk diffusion test. n.d.: not determined. Data represents mean ± standard deviation of experiment realized in triplicate.

**Table 3:** Antileishmanial activity against *Leishmania amazonensis* and cytotoxicity against BALB/c peritoneal macrophages of *Cinnamomum zeylanicum*,* Curcuma longa*, and *Origanum vulgare* essential oils.

Essential oils/ compounds	*L. amazonensis* IC_50_		Cytotoxicity CC_50_	SI
	Promastigote (*µ*g/mL)	Intracellular amastigote (*µ*g/mL)	Peritoneal macrophage (*µ*g/mL)	

*Cinnamomum zeylanicum*	>500 ^a,b,c^	n.d.	>1000	n.d.
*Curcuma longa*	308.4 ± 1.402 ^a,d,e^	63.3 ± 1.369 ^a^	>1000	>15.79
*Origanum vulgare*	405.5 ± 1.119 ^b,d,f^	n.d.	>1000	n.d.
Amphotericin B	0.764 ± 0.139 ^c,e,f^	1.045 ± 0.145 ^a^	37.22 ± 1.834	35.6

Data represents mean ± standard deviation of at least two experiments realized in triplicate. CC_50_: cytotoxic concentration for 50% of cells; IC_50_: inhibitory concentration for 50% of parasites; SI: selectivity index; n.d.: not determined. Equal letters in the same column mean statistical difference between IC_50_ (p<0.0001) by one-way ANOVA and Tukey's multiple comparisons test.

## Data Availability

The data used to support the findings of this study are included within the article.

## Abbreviations

DMSO: Dimethyl sulfoxide
GC-MS: Gas chromatography-mass spectrometry analyses
CFU: Colony-forming unit
MIC: Minimum inhibitory concentration
BHI: Brain heart infusion
MTT: 3-(4,5-Dimethylthiazol-2-yl)-2,5-diphenyltetrazolium bromide
IC_50_: Inhibitory concentration of 50%
CC_50_: 50% cell cytotoxicity
SI: Selectivity index
NO: Nitric oxide
LPS: Lipopolysaccharide.
